# Pregnancy-Induced Thrombotic Microangiopathy in Systematic Lupus Erythematosus: A Case Report

**DOI:** 10.7759/cureus.52248

**Published:** 2024-01-14

**Authors:** Basil Alnasrallah, Eman Alabbad, Mohammed M Aljishi, Zainab A Alkhuraidah, Sumayah Alsabaa

**Affiliations:** 1 Nephrology, Qatif Central Hospital, Qatif, SAU; 2 Internal Medicine, Qatif Central Hospital, Qatif, SAU; 3 Pathology, Qatif Central Hospital, Qatif, SAU

**Keywords:** therapeutic plasma exchange (tpe), acute kidney injury(aki)), pregnancy-related complications, systemic lupus erythromatosus, thrombotic microangiopathy (tma)

## Abstract

Thrombotic microangiopathy (TMA) is a severe systemic disorder with multiorgan manifestations due to thrombosis of the microvasculature. Pregnancy and post-partum are particularly high-risk periods for many forms of TMA. The disease progression is rapid and can lead to organ failure and even death; therefore, urgent recognition and treatment are paramount. The presence of other triggers such as infections or autoimmune diseases like systematic lupus erythematosus (SLE) can add further complexity, which emphasizes the need for definitive diagnostic investigations such as kidney biopsy to promptly direct further diagnosis and management. We describe a case of a 27-year-old female with post-partum severe acute kidney injury and nephrotic range proteinuria. She had a new diagnosis of active SLE and was found to have TMA on kidney biopsy without conclusive features of lupus nephritis. She was managed successfully with plasma exchange with rapid improvement of her kidney markers.

## Introduction

Thrombotic microangiopathy (TMA) is a serious pathological state where there is microvascular thrombosis leading to the mechanical destruction of red blood cells and consumption of platelets resulting in microangiopathic hemolytic anemia (MAHA) and thrombocytopenia, respectively [1]. The thrombotic occlusion of the small blood vessels preferentially affects the kidneys, brain, and heart, and this leads to the organ's dysfunction with significant morbidity and mortality [1,2]. The systemic process of TMA is coupled with a number of biochemical findings such as thrombocytopenia due to platelet aggregation and thrombi formation, anemia and presence of schistocytes due to fragmentation of red blood cells, raised lactate dehydrogenase (LDH) due to tissue ischemia and cell lysis, and low plasma haptoglobin due to intravascular hemolysis as it binds to free hemoglobin [3].

Several causes for TMA have been reported, both hereditary and acquired [1]. Pregnancy and the postpartum period are well-recognized triggers for TMA and it is thought that this is due to the increase in the production of Von Willebrand factor (VWF), which in turn increases consumption of ADAMTS13 with subsequent thrombosis [3]. The spectrum of pregnancy-associated TMAs includes disorders that are relatively common and cause secondary forms of TMA, such as pre-eclampsia, eclampsia, and HELLP (hemolysis, elevated liver enzymes, and low platelets) syndrome. These disorders are part of the same syndrome with different presentations and severity [4]. In addition, autoimmune conditions, such as systematic lupus erythematosus (SLE) and catastrophic antiphospholipid syndrome (CAPS) can also present with TMA. The activation of both classical and alternative complement pathways appears to play key roles in the SLE-associated TMA [3]. During pregnancy, other serious but less common causes of TMAs are hemolytic uremic syndrome (HUS), thrombotic thrombocytopenic purpura (TTP), and atypical hemolytic uremic syndrome (aHUS).

The clinical presentation of TMA in pregnancy is challenging and the presence of active autoimmune disorders such as SLE at the same time can make it a diagnostic dilemma. We present a case of severe acute kidney injury (AKI) due to TMA in a young female with active SLE in pregnancy and the postpartum period.

## Case presentation

A 27-year-old female, gravida 5, para 4, with no history of illness prior to her last pregnancy in 2021, presented in the third trimester with arthritis, headache, and generalized fatigue. The patient was found to have hypertension, renal impairment, and proteinuria. She was managed as pre-eclampsia with antihypertensives and had an early induction of vaginal delivery at 32 weeks of gestation. Her labs revealed antinuclear antibodies (ANA) +4, positive anti-double strand DNA, serum creatinine of 140 mmol/L, low complements' levels, 24-hour urine protein of 4377 mg, erythrocyte sedimentation rate (ESR) of 135 mm per hour, and C-reactive protein (CRP) of 9.5 mg/dl. At that point, she was diagnosed as SLE with likely lupus nephritis (LN) and started on methylprednisolone 1 gram IV for three doses, followed by oral prednisolone 1 mg/kg/day along with hydroxychloroquine 200 mg once daily. The kidney biopsy was deferred at that point due to the postpartum status.

After discharge, she presented again at 40 days postpartum on February 23, 2022, with pleuritic chest pain, dyspnea, generalized fatigue, and myalgia. Her labs showed hemoglobin of 5.7 g/dL, serum platelets of 105,000 per microliter, and serum creatinine of 283 mmol/L with an estimated glomerular filtration rate (eGFR) of 20 ml/minute/1.73 m^2^ by Chronic Kidney Disease Epidemiology Collaboration (CKD-EPI) equation, 24-hour urine protein 4972 mg, and low levels of complements 3 and 4 at 54.5 mg/dl and 10 mg/dl, respectively. During her admission, she was managed initially as a relapse of SLE with LN and was given methylprednisolone 1 g per day for four days, hydroxychloroquine 200 mg once daily, and mycophenolate mofetil 1500 mg twice a day. Interestingly, the makers of hemolysis were not elevated, the LDH was 213 unit/L, no schistocytes on blood film, and the total bilirubin was 2.5 µmol/L. Unfortunately, ADAMTS13 activity level, complement antibody, and gene mutation testing were not available at that time.

**Figure 1:**
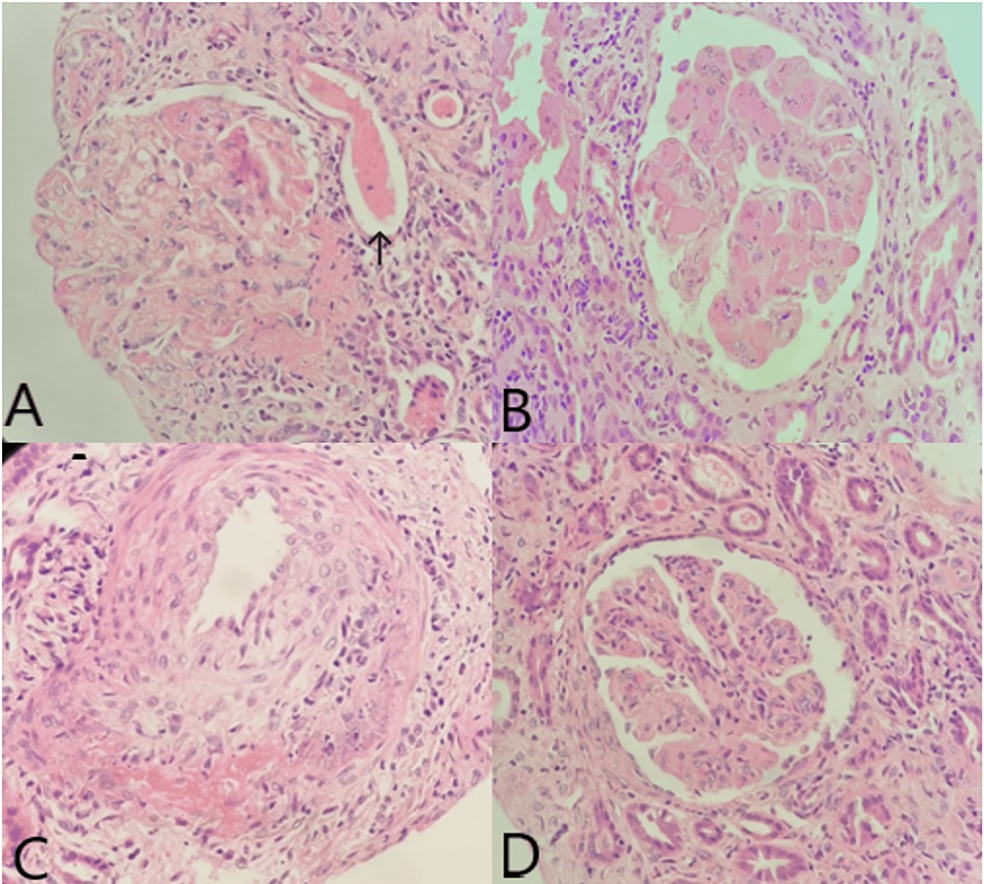
First kidney biopsy (A) The glomerular vascular pole is showing fibrinoid necrosis. A thrombus is also seen in one capillary lumen. The glomerulus has fibrillary appearance with duplication of the glomerular capillary basement membrane (H&E stain, 400x). (B) The glomerulus is showing mesangiolysis and the capillary lumina are occluded by homogenous eosinophilic thrombi (H&E stain, 400x). (C) There is fibrinoid necrosis with apoptotic bodies in the wall of this interlobular artery (H&E stain, 400x). (D) Few glomeruli are showing endocapillary hypercellularity that is associated with karyorrhexis (H&E stain, 400x)

Her kidney tests remained markedly deranged despite the initial therapy; therefore, we proceeded with kidney biopsy (Figure 1) which revealed several glomeruli showing capillary lumina filled with thrombi, double contour of glomerular capillary basement membrane, and mesangiolysis. Arteriolar and arterial fibrinoid necrosis associated with apoptotic bodies was seen. These features were consistent with thrombotic microangiopathy. Also, there were few glomeruli with endocapillary hypercellularity that were suggestive of LN; however, the direct immunofluorescence study demonstrated only mesangial granular deposit for complement C3 and no deposit for all the immunoglobulins. Therefore, 10 days after commencing the treatment for presumed LN with maximum immunosuppression as described above, we elected to manage the patient as postpartum TMA and to commence her on plasma exchange; her serum creatinine remained markedly elevated at 252 mmol/L at that point. The patient received plasmapheresis with 1.5 plasma volume of fresh frozen plasma on alternate days with significant improvement of her serum creatinine from 252 to 80 mmol/L. After completing four sessions, the 24-hour urine protein improved from 4972.8 mg to 2978 mg and reached 1288 mg within a month. 

**Figure 2:**
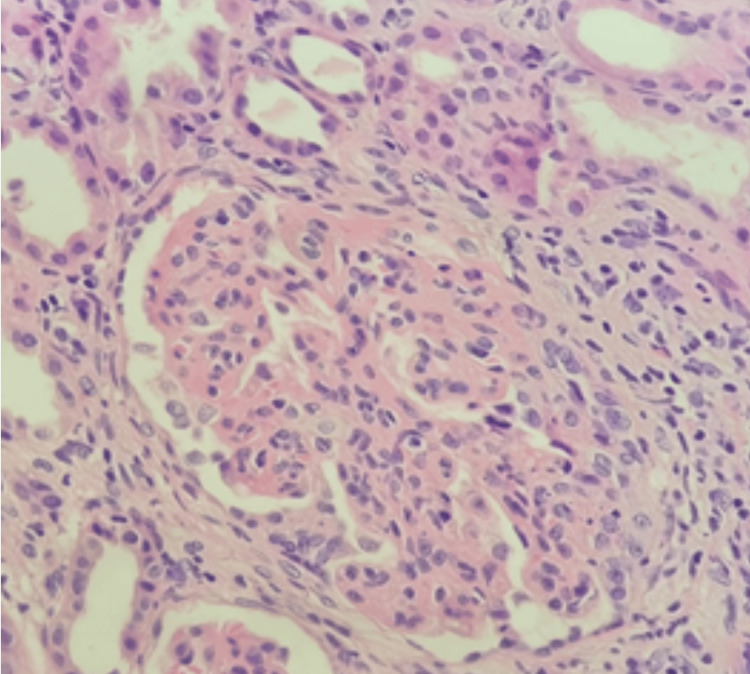
Second kidney biopsy Features of lupus nephritis with endocapillary hypercellularity that is associated with karyorrhexis and wire loop lesion. No features of TMA are seen (H&E stain, 400x)

In the following year, the level of proteinuria increased to 11036.4 mg/24 hours and serum creatinine was 104 mmol/L. A new kidney biopsy was performed (Figure 2), and 17 out of 33 viable glomeruli showed endocapillary hypercellularity associated with karyorrhexis or leukocyte infiltration. Twelve glomeruli revealed global sclerosis and three glomeruli revealed cellular crescent. The direct immunofluorescence study demonstrates intense granular staining for all the immunoglobulin; IgG, IgA, IgM, and for the complements C3 and C1q mainly along the peripheral capillary wall. Some mesangial immune deposits were also noted in the C3 and the IgA. No features of TMA were seen. The biopsy was diagnosed as class III/IV LN.

**Figure 3:**
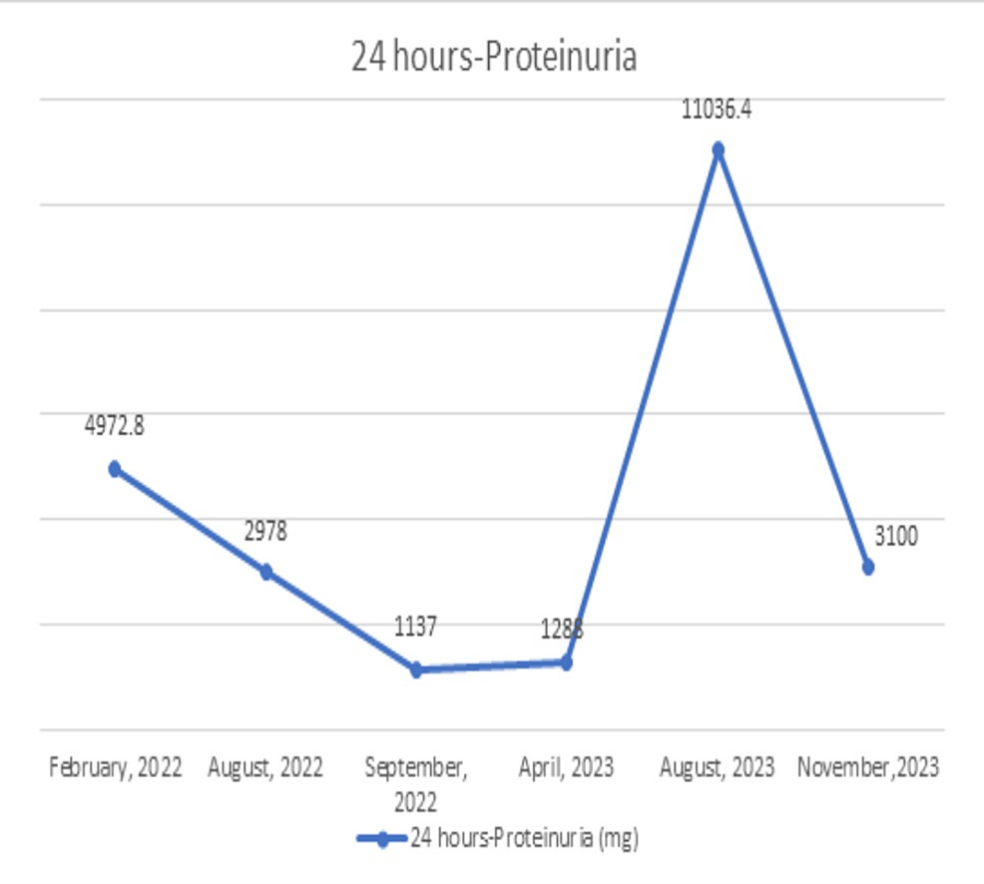
Change in proteinuria

**Figure 4:**
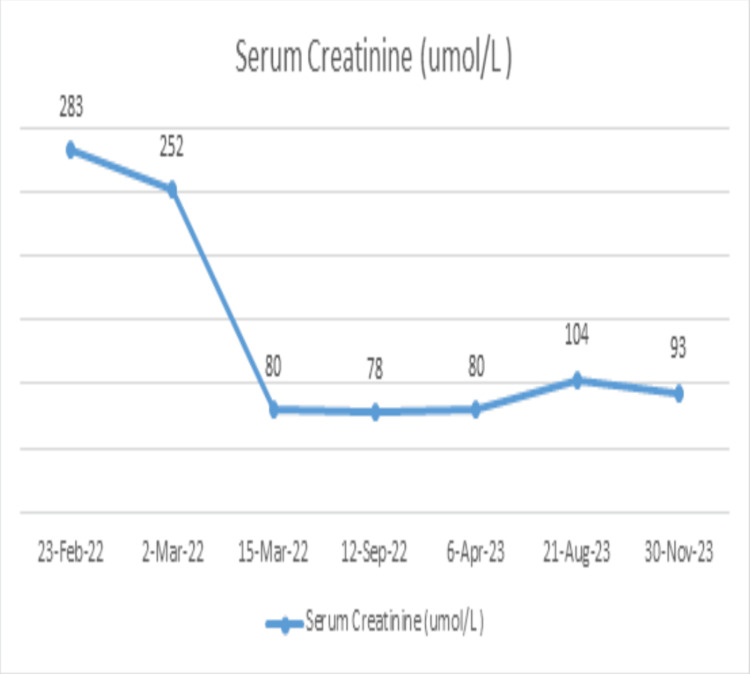
Change in serum creatinine

The patient was treated with a pulse of IV methylprednisolone 1000 mg for three days followed by oral prednisolone at 1 mg /kg/day for two months and mycophenolate mofetil increased again to 1500 mg twice a day. After that, the levels of proteinuria decreased to 3100 mg/24 hours and serum creatinine improved to 93 mmol/L three months later (Figures 3, 4). Her blood work during the two admissions post delivery is summarized in Table 1.

**Table 1:** Summary of blood tests C3: Complement 3; C4: Complement 4

Test	First admission post delivery		Second admission post delivery	
	March 1, 2022	March 10, 2022	August, 2023	November,2023
Hemoglobin (g/dL)	6.17	7.37	13.1	14.5
White blood count (/uL)	6.97 ´10^3^	4.53 ´10^3^	9.01 ´10^3^	7.32 ´10^3^
Platelets (/uL)	105 ´10^3^	129 ´10^3^	248 ´10^3^	217 ´10^3^
Red blood count (/uL)	2.29 ´10^6^	2.61 ´10^6^	4.93 ´10^6^	5.4 ´10^6^
Lactate dehydrogenase (U/L)	226	266		
Serum creatinine (umol/L)	252	91	104	72
Blood urea nitrogen (umol/L)	17.2	15.3	8	5.4
Sodium (mmol/L)	136	136	141	138
Potassium (mmol/L)	5.23	3.5	4.17	3.75
C3 (mg/dL)	60.5	56.7	81.9	
C4 (mg/dL)	13.4	10.7	12.4	

## Discussion

TMA is a serious systemic illness with progressively life-threatening thrombocytopenia, MAHA, and renal dysfunction [5]. TMA can be classified as primary, characterized by a complement mutation or complement autoantibodies, such as TTP and aHUS, or secondary due to infections, pregnancy, and autoimmune disorders such as SLE [6]. During pregnancy, the different causes of TMA have common clinical and laboratory findings which make it challenging to distinguish them apart. The frequency of the disorder and the timing of the presentation can serve as practical clues, as pre-eclampsia or HELLP syndrome have a relatively high incidence of one per 20 and one per 1000 pregnancies, respectively, while syndromes of primary TMA such as HUS or TTP are much less common at one per 25000 and one per 200000 pregnancies, respectively [7]. In addition, pregnancy-associated HUS is the only form of TMA to occur most frequently in the postpartum period and up to three months post-delivery in almost three-fourths of cases, and TMA starting in the postpartum of an uneventful pregnancy is very suggestive of complement-mediated aHUS [8,9]. Moreover, checking the activity testing of ADAMTS13 can be diagnostic in pregnancy-associated TTP [10]. However, this should be done prior to commencing the plasma exchange as the activity level of the test will change significantly and correspond to the clinical improvement once the therapy is started [11]. Unfortunately, testing might not always be available or requires sending to expert reference centers, which can extend the timing for diagnosis to days or even weeks. The PLASMIC (Platelet count; combined hemoLysis variable; absence of Active cancer; absence of Stem-cell or solid-organ transplant; Mean corpuscular volume; International normalised ratio; Creatinine) score has been developed to assist clinicians in deciding the likelihood of severe ADAMTS13 deficiency when the result of ADAMTS13 is not available [12]. In our case, the targeted testing was not done prior to the plasma exchange as there were no clear systemic signs of TMA. This highlights the need for clinicians to be aware of this diagnosis in this cohort of patients. The calculated PLASMIC score was 4 points, which is considered low and gives a 0% risk of severe ADAMTS13 deficiency. However, this didn't delay the management with plasma exchange which eventually resulted in a good recovery.

The kidney biopsy showed clear evidence of TMA with glomerular capillaries filled with thrombi; however, different forms of TMA are often indistinguishable based on the kidney biopsy findings. Moreover, immunofluorescence is usually negative apart from positive staining for fibrinogen with glomerular capillaries, arterioles, and small arteries [13]. In our case, although the first biopsy had granular C3 staining, this was felt to be non-specific as only a few reports have signaled that immunostaining might indicate complement activation in TMA. In addition, no specific or sensitive markers of complement activation are yet known for this entity [14].

For some forms of pregnancy-associated TMA such as pre-eclampsia, eclampsia, and HELLP syndrome, the rapid delivery can be sufficient to control the disorder and for other forms such as TTP and HUS, it can help achieve more rapid remission [10]. Other lines of management such as plasma exchange should be considered especially when there is an atypical presentation of pregnancy-associated TMAs, life-threatening neurological or cardiac findings, or profound thrombocytopenia (<30g/L) [10]. Expectant management with close monitoring would be reasonable if improvement in hemolysis markers, platelet levels, and no deterioration of renal function. If aHUS diagnosis is made by exclusion of other possibilities, then anti-C5 monoclonal antibodies should be initiated instead of plasma exchange [10]. Although the safety of the use of anti-c5 treatment in pregnancy has not been assessed in controlled clinical trials, the limited initial data suggest its safety, especially when considering the potentially catastrophic effects of uncontrolled TMA in pregnancy. Renal TMA can happen in the context of active LN and plays an important role in its natural history [6]. In SLE, the histopathological presence of TMA in the kidneys is a hallmark of severe and active renal disease with worse outcomes [15,16].

In this case, the presence of SLE and makers of activity both biochemically and clinically brought about an even bigger challenge as it can also provide a potential trigger for TMA even without clear systemic markers of hemolysis. The finding of TMA on the renal biopsy without conclusive LN was helpful in changing the route of management, especially as the renal clinical abnormalities were persisting prior to the plasma exchange.

## Conclusions

TMA in pregnancy and the postpartum period is a complex and serious disorder that requires a high index of suspicion and a prompt course of action. Other coexisting elements such as autoimmune disorders or infections can make the diagnosis a real challenge. The natural history of the illness especially in relation to delivery along with targeted testing can aid the diagnosis and management. Histopathological investigations can provide very valuable information and should be pursued, especially when renal involvement is suspected.

## References

[1] Aviner S, Bibi H (2014). Syndromes of thrombotic microangiopathy. N Engl J Med.

[2] Fakhouri F, Zuber J, Frémeaux-Bacchi V, Loirat C (2017). Haemolytic uraemic syndrome. Lancet.

[3] Thompson GL, Kavanagh D (2022). Diagnosis and treatment of thrombotic microangiopathy. Int J Lab Hematol.

[4] Mol BWJ, Roberts CT, Thangaratinam S, Magee LA, de Groot CJM, Hofmeyr GJ (2016). Pre-eclampsia. Lancet.

[5] Figueiredo CR, Escoli R, Santos P, Sofia F, Lopes K (2022). Thrombotic microangiopathy in a patient with systemic lupus erythematosus and anti-factor H autoantibodies. CEN Case Rep.

[6] Kotb HA, Mokbel A, Elmaghraby AA, Fadda S (2016). Thrombotic microangiopathy in lupus nephritis patients. Kasr Al Ainy Medical Journal.

[7] Scully M (2016). Thrombotic thrombocytopenic purpura and atypical hemolytic uremic syndrome microangiopathy in pregnancy. Semin Thromb Hemost.

[8] Huerta A, Arjona E, Portoles J (2018). A retrospective study of pregnancy-associated atypical hemolytic uremic syndrome. Kidney Int.

[9] Fakhouri F, Roumenina L, Provot F (2010). Pregnancy-associated hemolytic uremic syndrome revisited in the era of complement gene mutations. J Am Soc Nephrol.

[10] Fakhouri F, Scully M, Provôt F (2020). Management of thrombotic microangiopathy in pregnancy and postpartum: report from an international working group. Blood.

[11] Zheng XL, Kaufman RM, Goodnough LT, Sadler JE (2004). Effect of plasma exchange on plasma ADAMTS13 metalloprotease activity, inhibitor level, and clinical outcome in patients with idiopathic and nonidiopathic thrombotic thrombocytopenic purpura. Blood.

[12] Bendapudi PK, Hurwitz S, Fry A, Marques MB, Waldo SW, Li A (2017). Derivation and external validation of the PLASMIC score for rapid assessment of adults with thrombotic microangiopathies: a cohort study. Lancet Haematol.

[13] Sethi S, Fervenza FC (2014). Pathology of renal diseases associated with dysfunction of the alternative pathway of complement: C3 glomerulopathy and atypical hemolytic uremic syndrome (aHUS). Semin Thromb Hemost.

[14] Kim YJ (2022). A new pathological perspective on thrombotic microangiopathy. Kidney Res Clin Pract.

[15] Song D, Wu LH, Wang FM (2013). The spectrum of renal thrombotic microangiopathy in lupus nephritis. Arthritis Res Ther.

[16] Gharbi C, Bourry E, Rouvier P, Hacini S, Letaief A, Baumelou A, Izzedine H (2010). Rapidly progressive lupus nephritis and concomitant thrombotic microangiopathy. Clin Exp Nephrol.

